# Fabrication of Metal Nanoparticles from Fungi and Metal Salts: Scope and Application

**DOI:** 10.1186/s11671-016-1311-2

**Published:** 2016-02-24

**Authors:** Khwaja Salahuddin Siddiqi, Azamal Husen

**Affiliations:** Department of Chemistry, Aligarh Muslim University, Aligarh, 202002 Uttar Pradesh India; Department of Biology, College of Natural and Computational Sciences, University of Gondar, P.O. Box #196, Gondar, Ethiopia

**Keywords:** Green synthesis, Metal nanoparticles, Antimicrobial, Fungi, Plant

## Abstract

Fungi secrete enzymes and proteins as reducing agents which can be used for the synthesis of metal nanoparticles from metal salts. Large-scale production of nanoparticles from diverse fungal strains has great potential since they can be grown even in vitro. In recent years, various approaches have been made to maximize the yield of nanoparticles of varying shape, size, and stability. They have been characterized by thermogravimetric analysis, X-ray diffractometry, SEM/TEM, zeta potential measurements, UV-vis, and Fourier transform infrared (FTIR) spectroscopy. In this review, we focus on the biogenic synthesis of metal nanoparticles by fungi to explore the chemistry of their formation extracellularly and intracellularly. Emphasis has been given to the potential of metal nanoparticles as an antimicrobial agent to inhibit the growth of pathogenic fungi, and on other potential applications.

## Review

### Introduction

Of all the processes developed so far, the fabrication of metal nanoparticles by the biogenic methods employing plant extract are more popular, innocuous, inexpensive, and environmentally friendly as they do not leave hazardous residues to pollute the atmosphere [1–6]. Chemical methods for the synthesis of nanoparticles are common, but their use is limited. The biogenic synthesis is, therefore, the best choice where inherently benign organic molecules do not pose a threat to human health and atmosphere. Microbes have a promising role in the fabrication of nanoparticles due to their natural mechanism for detoxification of metal ions through reduction that can be achieved extra- or intracellularly by bioaccumulation, precipitation, biomineralization, and biosorption [4, 7–12].

Use of microgranisms in the green synthesis of metal nanoparticles with special reference to the precious metals using fungi has been done [10, 13–17]. Since fungi contain enzymes and proteins as reducing agents, they can be invariably used for the synthesis of metal nanoparticles from their salts. Since some fungi are pathogenic, one has to be cautious while working with them during experiment. Fungus biomass normally grows faster than those of bacteria [18] under the same conditions. Although synthesis of metal nanoparticles by bacteria is prevalent, their synthesis by fungi is more advantageous [19] because their mycelia offer a large surface area for interaction. Also, the fungi secrete fairly large amount of protein than bacteria; therefore, the conversion of metal salts to metal nanoparticles is very fast.

Engineered metal nanoparticles of varying size and shape from the diverse fungal species and yeast are listed in Table 1. Extracellular synthesis of nanoparticles involves the trapping of the metal ions on the surface of the cells and reducing them in the presence of enzymes, while intracellular synthesis occurs into the fungal cell in the presence of enzymes. Fungi secrete extracellular proteins which have been used to remove metal ions as nanoparticles. In a broad sense, the metal nanoparticles can be extensively used in different areas of agriculture and technology [2, 5, 6, 20, 21]. Many metal nanoparticles are antibacterial and find extensive uses in medicine [6, 22–24]. The antibacterial efficiency is enhanced manifold when a nanoparticle of one metal is coupled with another such as those of copper and silver. Although in recent times several organisms have been investigated for the fabrication of nanoparticles, its mechanism is still not well understood. This review, therefore, focuses on the biogenic synthesis of metal nanoparticles by fungi and attempts to explore the chemistry of their formation extracellularly and intracellularly.

**Table 1:** Engineered metal nanoparticles of varying size and shape fabricated from fungal and yeast species

Fungi and Yeast	Nanoparticles	Size (nm)	Shape	Location	References
*Alternaria alternate*	Au	12 ± 5	Spherical, triangular, hexagonal	Extracellular	[25]
*Aspergillus clavatus*	Au	24.4 ± 11	Triangular, spherical and hexagonal	Extracellular	[26]
*A. flavus*	Ag	8.92	Spherical	Cell wall	[27]
*A. fumigatus*	ZnO	1.2–6.8	Spherical and hexagonal	Extracellular	[28]
	Ag	–	–	Extracellular	[29]
*A. niger*	Au	12.8 ± 5.6	Spherical, elliptical	–	[30]
	Au	10–20	Polydispersed	Extracellular	[31]
*A. oryzae* TFR9	FeCl_3_	10–24.6	Spherical	–	[32]
*A. oryzae* var. *viridis*	Au	10–60	Various shapes (cell-free filtrate), mostly spherical (biomass)	Mycelial surface	[33]
*A. sydowii*	Au	8.7–15.6	Spherical	Extracellular	[34]
*A. terreus*	Ag	1–20	Spherical	Extracellular	[35]
*A. tubingensis*	Ca_3_P_2_O_8_	28.2	Spherical	Extracellular	[36]
*Aureobasidium pullulans*	Au	29 ± 6	Spherical	Intracellular	[37]
*Candida albicans*	Au	5	Monodispersed spherical	Cell-free extract	[38]
	Au	20–40	Spherical	–	[39]
		60–80	Non spherical		
*C. glabrata*	CdS	20 Å, 29 Å	Hexamer	Intra- and extracellular	[40]
	CdS	–	–	Intracellular	[41]
*Cladosporium cladosporioides*	Ag	10–100	Spherical	–	[42]
*Colletotrichum* sp.	Au	8–40	Spherical	Mycelial surface	[43]
*Coriolus versicolor*	Au	20–100, 100–300	Spherical and ellipsoidal	Intra- and extracellular	[44]
	Ag	25–75, 444–491	Spherical	Intra- and extracellular	[45]
*Cylindrocladium floridanum*	Au	19.05	Spherical	Extracellular	[46]
	Au	5–35	Spherical	Outer surface of the cell wall	[47]
*Epicoccum nigrum*	Au	5–50	–	Intra- and extracellular	[48]
*Fusarium oxysporum*	Pt	70–180	Rectangular, triangular, spherical and aggregates	–	[49]
	CdS	–	–	Extracellular	[50]
	Ag	–	–	Extracellular	[51]
	Ag	20–50	Spherical	Extracellular	[14]
	Au	2–50		-	[52, 53]
	Au	8–40	Spherical, triangular	Extracellular	[54]
	PbCO_3_, CdCO_3_	120–200	Spherical	Extracellular	[55]
	SrCO_3_	10–50	Needlelike	Extracellular Extracellular	[56]
	CdSe	9–15	Spherical	Extracellular	[57]
	CdS	5–20	Spherical	Extracellular	[58]
	TiO_2_	6–13	Spherical	Extracellular	[59]
	BaTiO_3_	4–5	Spherical	Extracellular	[60]
	ZrO_2_	3–11	Spherical		[61]
*F. semitectum*	Au	10–80	Spherical	Extracellular	[62]
*Hansenula anomala*	Au	14	–	–	[63]
*Helminthosporum solani*	Au	2–70	Spheres, rods, triangles, pentagons, pyramids, stars	Extracellular	[64]
*Hormoconis resinae*	Au	3–20	Spherical	Extracellular	[65]
*Macrophomina phaseolina*	Ag	5–40	Spherical	Cell-free filtrate	[144]
*Neurospora crassa*	Au	32 (3–100)	Spherical	Intracellular	[66]
*Pediococcus pentosaceus*	Ag	–	–	Extracellular	[67]
	Au	–	–	Intracellular	[68]
*Penicillium brevicompactum*	Au	10–60	Spherical, triangular and hexagonal	Extracellular	[69]
*P. fellutanum*	Ag	5–25	Spherical	Extracellular	[70]
*P. nagiovense* AJ12	Ag	25 ± 2.8	Spherical	Cell-free filtrate	[15]
*P. rugulosum*	Au	20–80	Spherical, triangular, exagonal	–	[71]
		20–40	Spherical		
*Penicillium* sp. 1–208	Au	30–50	Spherical	Cell filtrate	[72]
*Phanerochaete chrysosporium*	Au	10–100	Spherical	Extracellular	[73]
*Phoma glomerata*	Ag	60–80	Spherical	–	[74]
*Pichia jadinii*	Au	<100	Spherical	Cytoplasm	[7]
*Pleurotus sajor caju*	Ag	30.5	Spherical	Extracellular	[75]
*Rhizopus oryzae*	Au	16–25	Spherical	Cell-free filtrate	[76]
*Saccharomyces cerevisiae*	Au	15–20 30	Spherical	Cell wall Cytoplasm	[77]
*Schizosaccharomyces pombe*	CdS	18 Å, 29 Å	–	Intra- and extracellular	[40]
*S. pombe*	CdS	1–1.5	Hexagonal	Intracellular	[78]
*S. pombe*	CdS			Intracellular	[79]
*Sclerotium rolfsii*	Au	25.2 ± 6.8	Spherical		[80]
*Trichoderma asperellum*	Ag	13–18	Nanocrystalline	Extracellular	[81]
*T. koningii*	Au	30–40	Small spheres to polygons	–	[82]
	Au	10–14	Spheres	Cell-free filtrate	[83]
*T. reesei*	Ag	5–50	–	Extracellular	[84]
*T. viride*	Ag	5–40	Spherical	Extracellular	[85]
*Verticillium* sp.	Au	–	–	Intracellular	[86]
*Verticillium* sp.	Au	20 ± 8	Spherical	Cell wall and cytoplasmic membrane	[87]
*V. volvacea*	Au	20–150	Triangular, spherical, hexagonal	–	[88]
*Yarrowia lipolytica* NCIM 3589	Au	15	Hexagonal, triangular	Associated with cell wall	[89]
*Y. lipolytica* NCIM 3589	Au		Various shape depending on Au^3+^ concentration	Intracellular	[90]

### Synthesis, Mechanism, and Characterization of Metal Nanoparticles

**Fig. 1:**
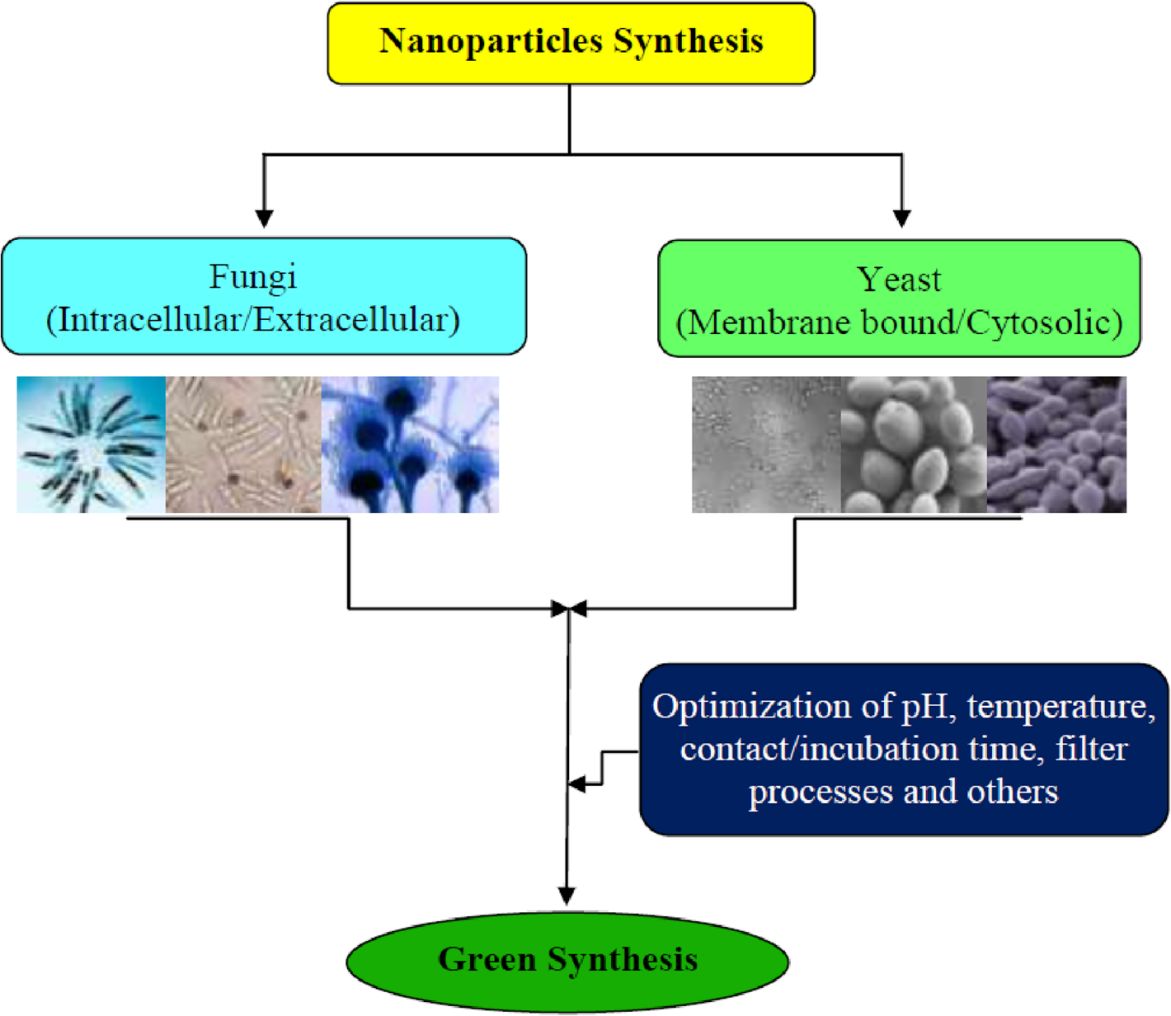
Synthesis of nanoparticles from fungi and yeast

**Fig. 2:**
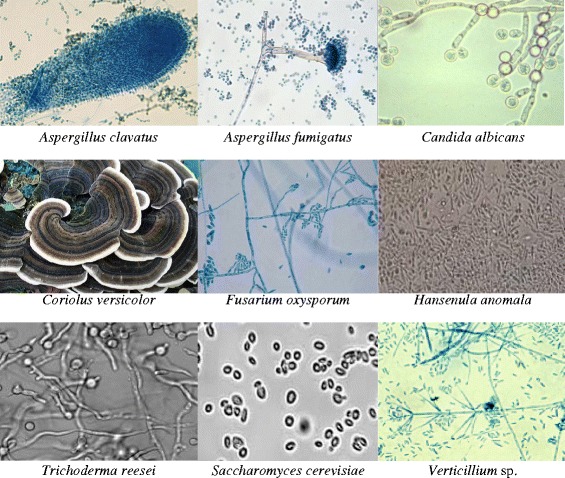
Frequently used fungi and yeasts for metal nanoparticle synthesis

Biogenic synthesis of metal nanoparticles involves bio-reduction of metal salts to elemental metal which may be stabilized by organic molecules present in the microbes such as fungi and bacteria. The other way of producing metal nanoparticles is biosorption where metal ions in the aqueous medium are bonded to the surface of the cell wall of the organisms. For large-scale production of nanoparticles, fungi and yeasts are preferred over other organisms (Figs. 1 and 2). When fungus is exposed to metal salts such as AgNO_3_ or AuCl_4_^−^, it produces enzymes and metabolites to protect itself from unwanted foreign matters, and in doing so, the metal ions are reduced to metal nanoparticles [91]. The fungi also produce napthoquinones and anthraquinones [92–95] which act as reducing agents. Thus, a specific enzyme can act on a specific metal. For instance, nitrate reductase is essential for ferric ion reduction to iron nanoparticles. It was reported that for metal ion reduction, not only the enzyme was necessary but also an electron shuttle [14]. It is well understood that nanomaterials may be beneficial or harmful to living systems [1–6]. For example, Cd, Hg, Pb, and Tl nanoparticles are toxic and produce adverse effect in mammals and plants. The toxicity also depends on their shape, size, and the nature of the specific metal ion.

#### Silver Nanoparticles

**Fig. 3:**
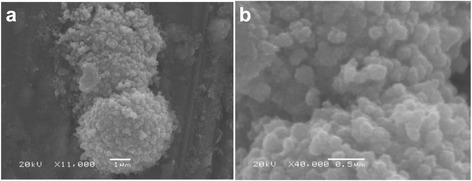
SEM image of the *Fusarium oxysporum* 07 SD strain at **a** ×11,000 and **b** ×40,000 magnification [14]

Major work has been done with silver nanoparticles produced by fungi extracellularly or intracellularly [29, 51]. The particle size is metal and fungi specific. The silver nanoparticles produced from the interaction of *Aspergillus fumigates* may not have the same dimension as those produced by *Fusarium oxysporum* even if the other conditions like concentration, pH, and temperature are identical [29]. The incubation time may also vary from 15 to 60 min [29, 51]. Synthesis of silver nanoparticles from *Trichoderma reesei* takes 72 h, but it is useful for large-scale production of nanoparticles. Their size ranges between 5 and 50 nm [84]. It has also been reported by Vahabi et al. [84] that manipulation of the method can produce enzymes up to 100 g/L which is unprecedented and requires confirmation. Silver nanoparticles of 20–50 nm, obtained from *F. oxysporum*, aggregate in spherical shape (Fig. 3) [14]. In this study, the extracellular reduction of metal ions was done by a nitrate-dependent reductase enzyme and a shuttle quinone. Sanghi et al. [45] studied the extra- and intracellular formation of silver nanoparticles by *Coriolus versicolor*, commonly known as white rot fungus. Extracellular production of silver nanoparticles from fungi *A. fumigates* [29] and *Phoma* sp. [96] has also been reported. In addition, the fungus, *Trichoderma viride*, was used to synthesize polydispersed silver nanoparticles of 5 to 40 nm at about 27 °C which showed an absorption band at 420 nm in UV-visible spectrum [85]. Antibacterial properties were tested against four bacterial strains namely, *Salmonella typhi* (gram-negative rods), *Escherichia coli* (gram-negative rods), *Staphylococcus aureus* (gram-positive cocci), and *Micrococcus luteus* (gram-positive cocci). It was observed that the antibacterial activities of ampicilin, kanamycin, erythromycin, and chloramphenicol were significantly enhanced in the presence of silver nanoparticles. *Geotricum* sp. was found to successfully produce silver nanoparticles with particle sizes ranging from 30 to 50 nm [97]. The fungus *Verticillium* (from *Taxus* plant) has also been used to synthesize silver nanoparticles with average size of 25 ± 12 nm at room temperature [87]. It is noteworthy that silver ions were not toxic to the fungal cells, and they continued to multiply even after biosynthesis of the silver nanoparticles. Rice husk is a cheap agro-based waste material, which harbors a substantial amount of silica in the form of amorphous hydrated silica grains. Therefore, it would be an ideal material to biotransform amorphous to crystalline silica nanoparticles. Yang et al. [98] have suggested that in such cases, the nanoparticles form complexes. It must be made clear at this stage that only metal ions are bonded to the organic groups by virtue of the positive charge on them and the lone pair of electrons on the organic functional groups. In no case may a neutral metal atom be bonded to any electron-donating molecule. There is always a great deal of confusion about metal ions and metal nanoparticles. A metal ion is a positively charged particle of much smaller size than an electrically neutral metal atom. When the metal ion is bonded to the surface of the fungal cell, it undergoes reduction to form metal nanoparticles with subsequent oxidation of organic molecules whether enzyme, protein, or peptide. It is quite obvious that oxidation and reduction are simultaneous processes.

Kowshik et al. [99] demonstrated the extracellular formation of 2- to 5-nm-long silver nanoparticles by a silver-tolerant yeast strain MKY3. Subramanian et al. [100] reported the effectiveness of marine yeasts (*Pichia capsulata*) derived from the mangrove sediments to synthesize silver nanoparticles (1.5 mM AgNO_3_, 0.3 % NaCl, pH 6.0, incubated at 5 °C for 24 h) that exhibited an absorption peak at 430 nm.

Extracellular biosynthesis and characterization of silver nanoparticles employing *Aspergillus flavus*, *A. fumigates*, *Neurospora crassa*, and *Phaenerochate chrysosporium* have been reported by many workers [29, 66, 101–103]. Nanoparticles of Au-Ag have also been reported [53, 54]. Gericke and Pinches [104] have obtained gold nanoparticles of different shapes and sizes from fungal cultures. It has been observed that their size can be controlled by monitoring concentration, pH, and temperature of the solution. It has also been noted that intracellular synthesis yields nanoparticles of smaller size.

The exact mechanism of intracellular synthesis of gold and silver nanoparticles is not known, but it is for sure that in the presence of fungi, they are formed on the surface of mycelia. It is proposed that the metal ions in the solution are attracted towards fungal mycelia by virtue of the positive charge on them and the slightly negative charge on the cell wall due to carboxylic groups on the enzyme or amino group of the protein, followed by reduction of the metal ions producing metal nanoparticles [105].

**Fig. 4:**
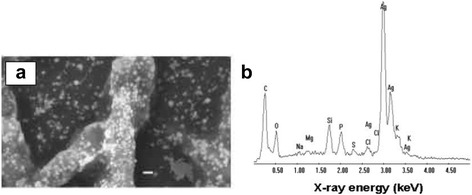
**a** SEM image of the *Verticillium* fungal cells after immersion in 10^−4^ M aqueous AgNO_3_ solution for 72 h (*scale bar* = 1 mm). **b** EDAX spectrum recorded from a film of fungal cells after formation of silver nanoparticles. Different X-ray emission peaks are labelled [106]

**Fig. 5:**
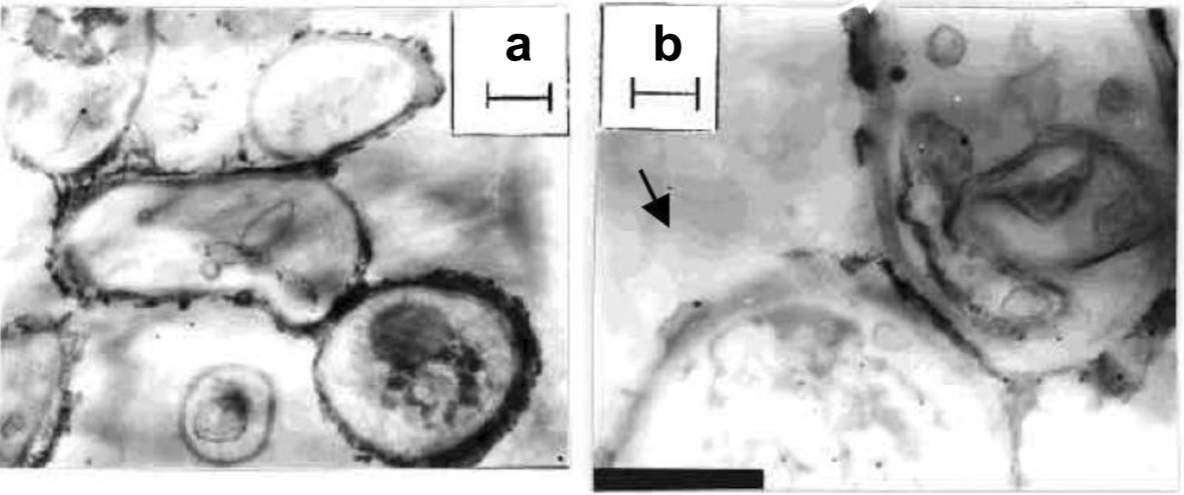
**a**, **b** TEM images of thin sections of stained *Verticillium* sp. cells after reaction with Ag+ ions for 72 h at different magnifications. *Scale bars* in **a** and **b** correspond to 1 and 500 nm, respectively [106]

The acidophilic fungus, *Verticillium* sp., isolated from the taxus plant, was allowed to interact with AgNO_3_ solution at 28 °C for 72 h. The transformation was monitored visually and spectroscopically by a change in color of the fungal biomass. Both gold and silver nanoparticle formation were further confirmed by a comparison of their spectra before and after their exposure to the fungi. It is also significant to note that the fungi keep on growing even after the formation of silver nanoparticles, indicating that they are not toxic to the *Verticillium* sp. However, in most of the bacterial species, the growth is arrested showing that Ag/Au nanoparticles are toxic to them. The inhibition of bacterial cell growth in the presence of Ag nanoparticles is assumed to be a defensive mechanism to sequester the metal ions as a consequence of which the Ag+ ions are reduced or complexed with proteins in the bacterial cells. The SEM image (Fig. 4a) of *Verticillium* spp. exposed to AgNO_3_ solution for 72 h showed uniform distribution of Ag nanoparticles over the entire surface of the fungal cell [106]. EDAX also indicated an abundance of Ag nanoparticles (Fig. 4b) besides other weak peaks for C, S, P, Mg, and Na. The TEM analysis of the above sample (Fig. 5a, b) displayed scattered dark spots identified as Ag nanoparticles of 25 ± 12 nm.

Li et al. have reported the fabrication of Ag nanoparticles of 1–20 nm from *Aspergillus terreus* in pretty good yields [35]. It is expected that the fungi secrete NADH as one of the components as reducing agents, which along with other ingredients, reduce the metal ions to metal nanoparticles. In order to confirm their hypothesis, NADH alone was added to AgNO_3_ solution which did not show any change in color. However, when NADH was added along with a fungal extract to AgNO_3_, the reaction started after a few minutes. It shows that NADH is a key factor in the synthesis of Ag nanoparticles, but other molecules are also essential which perhaps, catalyze the redox reaction. In many microorganisms, NADH is present as a coenzyme such as reductase secreted by *A. terreus*. Since NADH acts as an electron carrier and Ag+ ions as electron acceptor, reduction of Ag+ to Ag nanoparticle occurs [58, 106]. The Ag nanoparticles were examined for their antimicrobial activity. The results (Table 2) indicated that nanoparticles are broad spectrum antimicrobial agents. In some cases, they are effective even against fluconazole-resistant fungi [107].

**Table 2:** Size of the inhibition zone for AgNPs synthesized by *Aspergillus terreus* against the tested microorganisms [35]

Tested pathogenic organisms	Mean size of inhibition zone (mm)	
	Control	Test
*Candida albicans* (ATCC 90028)	9	16 ± 1
*C. krusei* (ATCC 6258)	10	14 ± 2
*C. parapsilosis* (ATCC 22019)	9	13 ± 1
*C. tropicalis* (JLCC 30394)	10	14 ± 1
*Aspergillus flavus* (IFM 55648)	9	13 ± 2
*A. fumigates* (IFM 40808)	9	14 ± 2
*Staphylococcus aureus* (ATCC 25923)	9	16 ± 1
*Pseudomonas aeruginosa* (ATCC 27853)	9	12 ± 1
*Escherichia coli* (ATCC 25922)	10	13 ± 1

*ATCC* American Type Culture Collection, USA; *IFM* Institute for Food Microbiology (at present the Medical Mycology Research Center, Chiba University), Japan; *JLCC* Culture Collection of Jilin University, Mycology Research Center, China

Control: AgNO3; test: AgNPs

#### Gold Nanoparticles

Biosynthesis of gold nanoparticles from fungi has been reviewed very recently [17]. They are resistant to oxidation and dispersed [107] nicely. The color corresponds to the particle size in general. For instance, yellow, red, and mauve refer to large, small, and fine nanoparticles, respectively, of varying size and morphology [108]. It is claimed that gold nanoparticles can be stabilized by substances like ascorbic acid and citrate [109]. Stabilization can also be achieved by polyvinyl alcohol [110]. Enzymes are said to be responsible for the biosynthesis of gold nanoparticles. The intra- or extracellular synthesis of nanoparticles by fungi is done in a simpler manner. The gold ions are trapped by the proteins and enzymes on the surface of the fungi and get reduced. They further form aggregates of large dimensions [111]. The gold nanoparticles synthesized from various sources have different properties. They have been checked for their cytotoxic effects against cancer [69]. Both the intracellular and extracellular reduction of AuCl or AuCl_3_ follow the same pathway [112]. Since AuCl requires one electron to give gold nanoparticles, it follows one-step reduction whereas AuCl_3_ requires three electrons and reduction occurs in three steps. As an example, when AuCl_3_ is dissolved in water, the following reactions occur at the mycelia of fungi which contain proteins, etc. and the metal nanoparticles are produced.

It is to be noted that in the event of intracellular gold nanoparticle formation, the Au^3+^ ions being smaller than Au^+^ ions penetrate or simply diffuse into the cell membrane and get reduced there. It is, however, inconclusive if diffusion of Au^3+^ ions into the fungal cell occurs through accumulation or absorption. As the concentration of gold nanoparticles increases, the Au^3+^/Au^+^ concentration falls. Metal nanoparticles induce oxidative stress in fungi and other microorganisms. Higher concentration of metal nanoparticles inhibits growth and protein expression [113] in *Rhizopus oryzae*. It is also likely that for a certain metal ion reduction, a specific type of protein is involved. However, it may be understood from hard and soft acid and base theory (HSAB) that donor acceptor complexation of metal with organic bases may occur.

Narayanan and Sakthivel [80] have demonstrated the formation of gold nanoparticles in the presence of the fungus *Cylindrocladium floridanum. They noted* that in 7 days, the fungi accumulated face-centered cubic (fcc) (111)-oriented crystalline gold nanoparticles on the surface of the mycelia. It was confirmed by the appearance of a characteristic peak at 540 nm in the UV-vis region. The nanoparticles are useful in degrading 4-nitrophenol where the process follows a pseudo-first-order kinetic model with the reaction rate constant of 2.67 × 10^−2^ m^−1^ with 5.07 × 10^−6^ mol dm^−3^ of gold of about 25 nm. As the reaction proceeds, an increase in gold nanoparticle concentration from 2.54 × 10^−6^ to 12.67 × 10^−6^ mol dm^−3^ occurs with a reduction in size from 53.2 to 18.9 nm.

Mukherjee et al. [87] have reported the formation of gold nanoparticles from *Verticillium* sp. which was found on the surface of mycelia. Gold nanoparticles have also been produced from *Verticillium* fungi. When HAuCl_4_ solution was added to fungal biomass, it started turning purple within a few hours of exposure while the aqueous solution of HAuCl_4_ remained colorless. It indicated intracellular nanoparticle formation. The morphology of Au nanoparticles does not appear to have a relationship with fungal species; as in all cases, several types of nanoparticles are formed [51, 87]. It was observed that old fungal biomass is less effective in producing Au nanoparticles than the fresh ones. It is probably due to a larger secretion of proteins and enzymes in the fresh fungal biomass than the aged ones.

Kumar et al. [64] showed the applicability of yeast species *Hansenula anomala* to reduce gold salt in the presence of amine-terminated polyamidoamine dendrimer as stabilizer. Lim et al. [114] used *Saccharomyces cerevisae* broth to synthesize gold and silver nanoparticles. Gold nanoparticles of 2- to 100-nm size were prepared at pH 4–6 in 24 h which had an absorption maximum at 540 nm. Extracellular synthesis of silver NP of 10–20 nm was done at pH 8–10 in 48 h. It displayed a characteristic absorption peak at 415 nm. Gold and silver nanoparticles, with face-centered cubic structures prepared from *Candida guilliermondii* [115], exhibited distinct surface plasmon peaks at 530 and 425 nm, respectively. These nanoparticles were tested against five pathogenic bacterial strains. The highest efficiency for both gold and silver nanoparticles was observed against *S. aureus*, which indicated the applicability of yeast-synthesized nanoparticles for environmental remediation. *Yarrowia lipolytica* was reported to be an effective reducing agent to produce gold nanoparticles and nanoplates by varying concentrations of chloroauric acid at pH 4.5 [90]. According to the findings, a mixture of 109 cells ml^−1^ and 0.5 or 1.0 mM of the gold salt developed a purple or golden red color indicating the formation of gold nanoparticles. Nanoparticles of different sizes were obtained by incubating 1010 cells ml^−1^ with 0.5, 1.0, or 2.0 mM chloroauric acid. It was confirmed that an increase in salt concentration at a fixed number of cells resulted in the increase of nanoparticles. On the other hand, an increase in cell numbers at a constant gold salt concentration resulted in a significant decrease in nanoparticle size. From Fourier transform infrared spectroscopy (FTIR) spectral data, the presence of carboxyl, hydroxyl, and amide groups on the cell surfaces was confirmed.

Soni and Prakash [116] have reported the green synthesis of gold nanoparticles from *Aspergillus niger* and identified it by a change in color and its absorption at 530 nm. They have also suggested that broadening of the band is due to the aggregation of gold nanoparticles. Perhaps it refers to the low concentration of the nanoparticles because the peak centered at 530 nm will obviously become sharp as a result of the increased quantity of nanoparticles. They have also reported that the Au nanoparticles are toxic to *Anopheles stephensi*, *Culex quinquefasciatus*, and *Aedes aegypti* mosquito larvae. Silver nanoparticles synthesized from *Pleurotus ostreatus* fungi were characterized by UV-vis, SEM, EDS, XRD, and TEM. Silver nanoparticles in solution were identified by the appearance of a peak at 440 nm in the visible region of the spectrum. XRD pattern showed their crystalline nature. The SEM and TEM images showed depressed Ag nanoparticles of nearly 50 nm. Their antimicrobial activity was tested against Gram-positive and Gram-negative bacteria namely, *E. coli*, *Klebsiella pneumonia*, *Pseudomonas aeruginosa*, *S. aureus*, and *Vibrio cholera*. It was observed that Ag nanoparticles are much less effective against the above pathogens relative to the antibiotics, but when antibiotics are fortified with Ag nanoparticles, their activity is enhanced. It is quite likely that a suitable mixture of antibiotic and Ag nanoparticles may be more effective as a medicine for drug-resistant pathogens.

#### Other Metal Nanoparticles

Castro-Longoria et al. [117] have produced platinum nanoparticles and their aggregates using the fungus *N. crassa*. Both intracellular single platinum nanoparticles of 4- to 3-nm diameter and spherical agglomerates of 20- to 110-nm diameter were produced. A comparison of platinum nanoparticles synthesized from biomass was made with those prepared from *N. crassa* extract. It was noticed that the platinum nanoparticles produced from the extract were only single crystal nano-agglomerates. However, the quantity of nanoparticles synthesized extracellularly differs significantly from those prepared intracellularly. Magnetite, Fe_3_O_4_ magnetite nanoparticles have been obtained from *F. oxysporum* and *Verticillium* sp. [118]. Extracellular synthesis of fairly smaller selenium nanoparticles of the order of 47 nm from *A. terreus* was also done in 60 min [119]. It was found that *Schizosaccharomyces pombe* and *Candida glabrata* were capable of intracellular production of CdS nanoparticles from cadmium salt in solution [120]. CdS nanoparticle synthesis using *S. pombe* has been considered to be dependent on a stress protein response [99]. Phytochelatin gets activated on exposure of *S. pombe* to cadmium and synthesizes phytochelatins. These phytochelatins chelate the cytoplasmic cadmium to phytochelatin–Cd complex. Thereafter, an ATP-binding cassette-type vacuolar protein transports phytochelatin–Cd complex across the vacuolar membrane. Within the vacuole, sulfide gets added to the complex to form a high-molecular-weight phytochelatin CdS^−2^ complex/CdS nanocrystal.

### Metal Nanoparticles and Plant Pathogenic Fungi

**Fig. 6:**
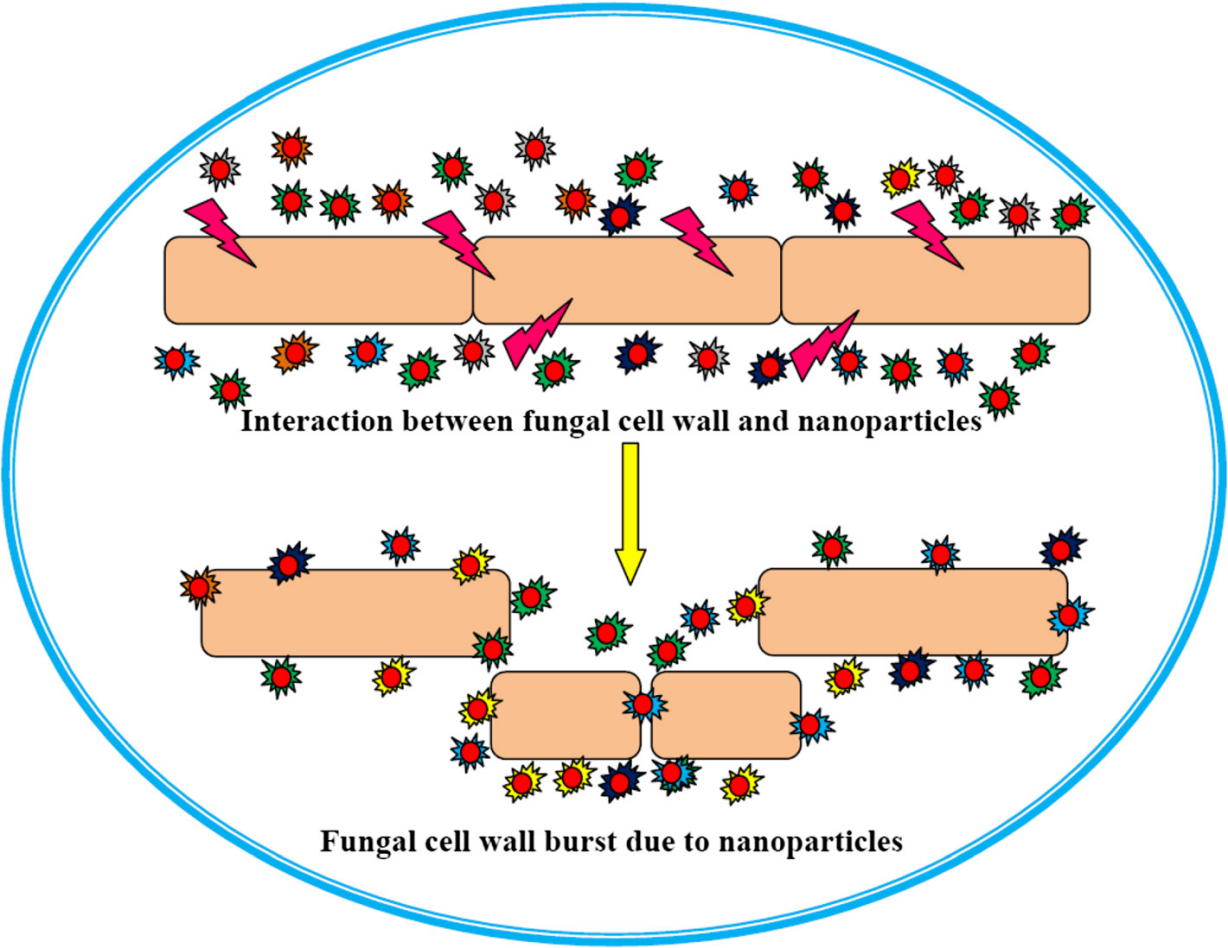
Possible mechanism behind fungus and nanoparticles interaction

Fungi are accountable for more than 70 % of all major crop diseases [121]. The annual crop losses due to pre- and post-harvest fungal diseases exceed 200 billion euros, and in the USA alone, over $600 million are annually spent on fungicides [122]. Impact of nanoparticles on crop plants is a rising area of research that needs to be meticulously explored. In recent years, engineered nanoparticles have achieved particular attention as a potential candidate for improving crop yield, resistance, and disease management technologies [5, 6, 123]. However, these applications are still in their infancy. This is simply due to the unprecedented and unforeseen health hazards and environmental concerns [6]. It is understood that the use of pesticides in agriculture is becoming more hazardous day by day. In order to replace such toxic materials by equally useful substances is an excellent choice, especially easily available silver nanoparticles which are antimicrobial for most of the fungal and bacterial diseases in man and plants [124, 125]. Jo et al. [126] have reported antifungal activity of Ag+ ions and Ag nanoparticles on pathogenic fungi. It is useful if a better protocol for its application in plants is developed. The Ag nanoparticles in aqueous medium catalyze complete destructive oxidation of microorganism [127]. Kim et al. [128] have studied the growth inhibition effect of three types of Ag nanoparticles against 18 different plant pathogenic fungi in vitro (Table 3). A variety of host plants including the cucumber family, tomato, potato, and cabbage, which are very prone to infections, have been treated. It has been noted that growth inhibition is concentration-dependent and the most effective concentration leading to complete destruction of fungi is 100 ppm. Possible mechanism of interaction between fungi and nanoparticles is presented in Fig. 6.

**Table 3:** List of plant pathogenic fungi (modified from 128)

Fungal species (KACC accession no.)	Common names	Host plants
*Alternaria alternata* (A-1 40019)	Alternaria leaf blight	Strawberry, pepper, tomato
*Alternaria brassicicola* (A-2 40857)	Black spot	Cauliflower, radish, cabbage, kale
*Alternaria solani* (A-3 40570)	Alternaria leaf spot	Pepper, tomato, eggplant, potato
*Botrytis cinerea* (B-1 40574)	Gray mold	Eggplant, tomato, potato, pepper, strawberry
*Cladosporium cucumerinum* (C-1 40576)	Scab	Eggplant, cucumber, pumpkin, melon
*Corynespora cassiicola* (C-9 40964)	Leaf spot	Pepper, cucumber, bean, tomato, sesame
*Cylindrocarpon destructans* (C-10 41077)	Root rot	Strawberry, ginseng, peony
*Didymella bryoniae* (D-1 40938)	Black rot	Cucumber, pumpkin, watermelon, melon
*Fusarium oxysporum* f. sp*. Cucumerinum* (F-1 40525)	Fusarium wilt	Cucumber
*F. oxysporum* f. sp. *Lycopersici* (F-2 40032)	Fusarium wilt	Tomato
*F. oxysporum* (F-3 40052)	Fusarium wilt	Tomato
*Fusarium solani* (F-4 41643)	Fusarium wilt	Potato, ginseng
*Fusarium* sp. (F-5 40050)	Fusarium rot	Potato, sweet potato, pepper, strawberry, pear tree
*Glomerella cingulata* (G-1 40895)	Anthracnose	Pepper, strawberry, grapevine
*Monosporascus cannonballus* M-1 40940)	Root rot	Cucumber, pumpkin, watermelon, melon
*Pythium aphanidermatum* (P-8 40156)	Damping-off	Tomato, tobacco, radish
*Pythium spinosum* (P-9 41060)	Root rot	Sweet potato, pumpkin, cabbage
*Stemphylium lycopersici* (S-3 40967)	Leaf spot	Eggplant, tomato, pepper

*KACC* Korean Agricultural Culture Collection, Suwon, Korea

Silver nanoparticles were used as an alternative to pesticides to control the sclerotia-forming phytopathogenic fungi [129]. The antifungal effect of doubly encapsulated silver nanoparticle solution against rose powdery mildew (leaf distortion, leaf curling, early defoliation, and reduced flowering) caused by *Sphaerotheca pannosa* var *rosae* was also studied [130]. A 10-ppm silver nanoparticle solution of 1.5 nm was sprayed over a large area infected by rose powdery mildew. After 2 days, more than 95 % of them faded out and did not recur for a week. The toxic effect of the silver nanoparticles of 5–24 nm on *Colletotrichum gloesporioides*, which causes anthracnose in a wide range of fruits, such as apple, avocado, mango, and papaya has also been studied [131]. A significant delay in growth of *C. gloesporioides* was observed. Silver nanoparticles may, therefore, be used as an alternative to fungicides for plant disease management. Das et al. [132] have reported extracellular synthesis of gold nanoparticles of 10 nm from *R. oryzae* which was employed for the generation of nanogold-bioconjugate structure. These nanostructures displayed excellent adsorption capacity and were successfully employed to purify water free from pathogens and pesticides.

### Application of Metal Nanoparticles

There are a myriad of applications of metal nanoparticles such as cosmetics, catalysts, lubricants, fuel additives, paints, agro-chemicals, food packaging, textile engineering, electronics, optics, environmental sensing, nanomedicine, drug and gene delivery agents, biodetection of pathogens, tumor destruction via heating (hyperthermia), magnetic resonance imaging, and phagokinetic studies [2–9, 23, 24, 133–136, 139]. Fungus-mediated synthesis of metal nanoparticles is getting much attention due to their extensive application in various sectors (Table 4). Durán et al. [141] have reported that silver nanoparticles (1.6 nm) obtained extracellularly from *F. oxysporum* can be incorporated in clothes which can prevent infection from *S. aureus*. Silver nanoparticles (1–10 nm) attach to the bacterial cell surface and significantly disrupt its respiration and permeability [154]. Silver nanoparticles (5–25 nm) from *A. fumigates* were produced to understand the biochemical and molecular mechanism of synthesis [29]. The ability of the fungi, *F. oxysporum*, to hydrolyze metal complexes demonstrates the formation of metal oxide semiconducting materials [59]. Namasivayam and Avimanyu [143] have reported that when the silver nanoparticles of 45–100 nm obtained from *Lecanicillium lecanii* were coated on the bleached cotton fabrics using acrylic binder, they became resistant to *S. aureus* and *E. coli* infection. A method was invented to decorate the growing fungal hyphae of *A. niger* with a high load of gold nanoparticles, which were initially produced using aqueous tea extract as a sole reducing/stabilizing agent [155]. Heat treatment of these hybrid materials yielded porous gold microwires. It is anticipated that the nanowire-based paper may be used to clean up oil and organic pollutants in water and soil sediments. Nanofibrous mats were prepared by Spasova et al. [156], which contained chitosan and *T. viride* spores. It was reported that *T. viride* kept at 28 °C grows much faster and fights for space and nutrients against *Fusarium* sp. and *Alternaria* sp. Moreover, *T. viride* produces extracellular hydrolytic enzymes which directly attack the pathogen and destroy their cell walls. Advances in luminescent nanocrystals have led to fluorescent labelling by QDs with bio-recognition molecules [157]. When *F. oxysporum* was incubated with a mixture of CdCl_2_ and SeCl_4_, highly luminescent CdSe quantum dots were produced at room temperature (26 ± 1 °C) [153]. In addition, nitrate reductase from *F. oxysporum* has been shown to catalyze the production of stable silver nanoparticles in vitro. It suggests the way for designing a rational enzymatic strategy for the synthesis of nanomaterials of different composition, shape, and size [158]. An optical sensor for the detection of pesticides (Siven 85 % wettable powder) in water using ZnCdSe QD films has also been developed [159].

**Table 4:** Applications of metal nanoparticles synthesized by fungi and yeasts

Nanoparticle	Fungi/yeasts	Application	References
Ag	*Alternaria alternata*	Enhancement in antifungal activity of fluconazole against *Phoma glomerata*	[136]
	*Aspergillus clavatus*	Antimicrobial activity	[137]
	*A. niger*	Antibacterial activity	[138]
	*A. niger*	Wound healing activity	[131]
	*Colletotrichum gloesporioides*	Antifungal activity	[141]
	*Fusarium acuminatum*	Antibacterial activity	[140]
	*F. oxysporum*	Textile fabrics	[141]
	*F. solani*	Textile fabric	[142]
	*Lecanicillium lecanii*	Textile fabrics	[143]
	*Macrophomina phaseolina*	Antimicrobial properties against multidrug-resistant bacteria	[144]
	*Penicillium oxalicum*	Catalytic activity	[145]
	*Penicillium* sp.	Antibacterial activity against MDR *E. coli* and *S. aureus*	[146]
	*Phytophthora infestans*	Antimicrobial activity	[147]
	*Pleurotus ostreatus*	Antimicrobial activity	[148]
	*Raffaelea* sp.	Antifungal activity	[149]
	*Trichoderma crassum*	Antimicrobial activity	[150]
	*T. viride*	Vegetable and fruit preservation	[151]
Au	*Aspergillus japonicus* AJP01	Catalytic activity	[152]
	*A. niger*	Toxic to mosquito larvae	[116]
	*Rhizopus oryzae*	Water hygiene management	[132]
Cds	*Saccharomyces pombe*	Electric diode	[78]
	*F. oxysporum*	Live cell imaging and diagnostics	[153]

## Conclusions

It is an established fact that biogenic synthesis of metal nanoparticles by fungi is a safe and economical process because stable and small-sized nanoparticles are generally produced. Their role in drug delivery, magnetic resonance imaging, catalysis, environmental sensing, textile engineering, food sectors and plant disease management is well known. Several precious metals may be easily recovered from large heap of wastes containing metal salts. This process of producing nanoparticles by a redox process may be employed to produce pure metals. The fungi may therefore be used in metallurgical operations to sequester metal from ores. It can save time and money. Since some of the metal ions are toxic to many microbes, they can be used as a prophylactic to inhibit their growth. However, a comprehensive protocol may be developed to control the morphology of metal nanoparticles for their application in all sectors of medicine, agriculture, and technology.

## References

[1] Song JY, Kim BS (2009). Rapid biological synthesis of silver nanoparticles using plant leaf extracts. Bioprocess Biosyst Eng.

[2] Nair R, Varghese SH, Nair BG, Maekawa T, Yoshida Y, Kumar DS (2010). Nanoparticulate material delivery to plants. Plant Sci.

[3] Rico CM, Majumdar S, Duarte-Gardea M, Peralta-Videa JR, Gardea-Torresdey JL (2011). Interaction of nanoparticles with edible plants and their possible implications in the food chain. J Agric Food Chem.

[4] Durán N, Marcato PD, Durán M, Yadav A, Gade A, Rai M (2011). Mechanistic aspects in the biogenic synthesis of extracellular metal nanoparticles by peptide, bacteria, fungi, and plants. Appl Microbiol Biotechnol.

[5] Husen A, Siddiqi KS (2014a) Carbon and fullerene nanomaterials in plant system. J Nanobiotechnol 12:16 10.1186/1477-3155-12-16 PMC4014205 24766786

[6] Husen A, Siddiqi KS (2014b) Phytosynthesis of nanoparticles: concept, controversy and application. Nano Res Lett 9:229 10.1186/1556-276X-9-229 PMC4031915 24910577

[7] Gericke M, Pinches A (2006). Microbial production of gold nanoparticles. Gold Bull.

[8] Li X, Xu H, Chen ZS (2011). Chen: Biosynthesis of nanoparticles by microorganisms and their applications. J Nanomater.

[9] Prabhu S, Poulose EK (2012). Silver nanoparticles: mechanism of antimicrobial action, synthesis, medical applications, and toxicity effects. Int Nano Lett.

[10] Husen A, Siddiqi KS (2014c) Plants and microbes assisted selenium nanoparticles: characterization and application. J Nanobiotechnol 12:28 10.1186/s12951-014-0028-6 PMC4274736 25128031

[11] Iravani S (2014). Bacteria in nanoparticle synthesis: current status and future prospects. Int Schol Res Notic.

[12] Singh R, Shedbalkar UU, Wadhwani SA, Chopade BA (2015). Bacteriagenic silver nanoparticles: synthesis, mechanism, and applications. App Microbiol Biotechnol.

[13] Sastry M, Ahmad A, Khan MI, Kumar R (2003). Biosynthesis of metal nanoparticles using fungi and actinomycete. Cur Sci.

[14] Durán N, Marcato PD, Alves OL, DeSouza G, Esposito E (2005). Mechanistic aspects of biosynthesis of silver nanoparticles by several *Fusarium oxysporum* strains. J Nanobiotechnol.

[15] Maliszewska I, Juraszek A, Bielska K (2014). Green synthesis and characterization of silver nanoparticles using ascomycota fungi *Penicillium nalgiovense* AJ12. J Clust Sci.

[16] Vágó A, Szakacs G, Sáfrán G, Horvath R, Pécz B, Lagzi I (2016). One-step green synthesis of gold nanoparticles by mesophilic filamentous fungi. Chem Phys Lett.

[17] Kitching M, Ramani M, Marsili E (2015). Fungal biosynthesis of gold nanoparticles: mechanism and scale up. Microb Biotechnol.

[18] Taherzadeh MJ, Fox M, Hjorth H, Edebo L (2003). Production of mycelium biomass and ethanol from paper pulp sulfite liquor by *Rhizopus oryzae*. Bioresour Technol.

[19] Pantidos N, Horsfall LE (2014). Biological synthesis of metallic nanoparticles by bacteria, fungi and plants. J Nanomed Nanotechnol.

[20] Mondal A, Basu R, Das S, Nandy P (2011). Beneficial role of carbon nanotubes on mustard plant growth: an agricultural prospect. J Nanopart Res.

[21] Alam MN, Roy N, Mandal D, Begum NA (2013). Green chemistry for nanochemistry: exploring medicinal plants for the biogenic synthesis of metal NPs with fine-tuned properties. RSC Adv.

[22] Kim JS, Kuk E, Yu KN, Kim JH, Park SJ, Lee HJ, Kim SH, Park YK, Park YH, Hwang CY, Kim YK, Lee YS, Jeong DH, Cho MH (2007). Antimicrobial effects of silver nanoparticles. Nanomed Nanotechno Biol Med.

[23] Sperling RA, Rivera Gil P, Zhang F, Zanella M, Parak WJ (2008). Biological applications of gold nanoparticles. Chem Soc Rev.

[24] Jelveh S, Chithrani DB (2011). Gold nanostructures as a platform for combinational therapy in future cancer therapeutics. Cancers.

[25] Sarkar J, Ray S, Chattopadhyay D, Laskar A, Acharya K (2012). Mycogenesis of gold nanoparticles using a phytopathogen *Alternaria alternata*. Bioprocess Biosyst Eng.

[26] Verma VC, Singh SK, Solanki R, Prakash S (2011). Biofabrication of anisotropic gold nanotriangles using extract of endophytic *Aspergillus clavatus* as a dual functional reductant and stabilizer. Nanoscale Res Lett.

[27] Vigneshwaran N, Ashtaputre NM, Varadarajan PV, Nachane RP, Paralikar KM, Balasubramanya RH (2007). Biological synthesis of silver nanoparticles using the fungus *Aspergillus flavus*. Mater Lett.

[28] Raliya R, Tarafdar JC (2013). ZnO nanoparticle biosynthesis and its effect on phosphorous-mobilizing enzyme secretion and gum contents in cluster bean (*Cyamopsis tetragonoloba* L.). Agirc Res.

[29] Bhainsa KC, D’Souza SF (2006). Extracellular biosynthesis of silver nanoparticles using the fungus *Aspergillus fumigatus*. Coll Surf B Biointerfaces.

[30] Bhambure R, Bule M, Shaligram N, Kamat M, Singhal R (2009). Extracellular biosynthesis of gold nanoparticles using *Aspergillus niger*—its characterisation and stability. Chem Eng Technol.

[31] Xie J, Lee JY, Wang DIC, Ting YP (2007). High-yield synthesis of complex gold nanostructures in a fungal system. J Phys Chem C.

[32] Raliya R (2013). Rapid, low-cost, and ecofriendly approach her for iron nanoparticle synthesis using *Aspergillus oryzae* TFR9. J Nanopart.

[33] Binupriya AR, Sathishkumar M, Vijayaraghavan K, Yun SI (2010). Bioreduction of trivalent aurum to nanocrystalline gold particles by active and inactive cells and cell free extract of *Aspergillus oryzae* var. *viridis*. J Hazard Mater.

[34] Vala AK (2015). Exploration on green synthesis of gold nanoparticles by a marine-derived fungus *Aspergillus sydowii*. Environ Prog Sustain Energy.

[35] Li G, He D, Qian Y, Guan B, Gao S, Cui Y, Yokoyama K, Wang L (2012). Fungus-mediated green synthesis of silver nanoparticles using *Aspergillus terreus*. Int J Mol Sci.

[36] Tarafdar JC, Raliya R, Rathore I (2012). Microbial synthesis of phosphorous nanoparticle from tri-calcium phosphate using *Aspergillus tubingensis* TFR-5. J Bionanosci.

[37] Zhang X, He X, Wang K, Yang X (2011). Different active biomolecules involved in biosynthesis of gold nanoparticles by three fungus species. J Biomed Nanotechnol.

[38] Ahmad T, Wani IA, Manzoor N, Ahmed J, Asiri AM (2013). Biosynthesis, structural characterization and antimicrobial activity of gold and silver nanoparticles. Colloids Surf B: Biointerfaces.

[39] Chauhan A, Zubair S, Tufail S, Sherwani A, Sajid M, Raman SC, Azam A, Owais M (2011). Fungus-mediated biological synthesis of gold nanoparticles: potential in detection of liver cancer. Int J Nanomedicine.

[40] Dameron CT, Reese RN, Mehra RK, Kortan AR, Carroll PJ, Steigerwald ML, Brus LE, Winge DR (1989). Biosynthesis of cadmium sulphide quantum semiconductor crystallites. Nature.

[41] Krumov N, Oder S, Perner Nochta I, Angelov A, Posten C (2007). Accumulation of CdS nanoparticles by yeasts in a fed-batch bioprocess. J Biotechnol.

[42] Balaji DS, Basavaraja S, Deshpande R, Mahesh DB, Prabhakar BK, Venkataraman A (2009). Extracellular biosynthesis of functionalized silver nanoparticles by strains of *Cladosporium cladosporioides* fungus. Coll Surf B Biointerfaces.

[43] Shankar SS, Ahmad A, Pasricha R, Sastry M (2003). Bioreduction of chloroaurate ions by geranium leaves and its endophytic fungus yields gold nanoparticles of different shapes. J Mater Chem.

[44] Sanghi R, Verma P (2010). pH dependant fungal proteins in the “green” synthesis of gold nanoparticles. Adv Mater Lett.

[45] Sanghi R, Verma P (2009). Biomimetic synthesis and characterisation of protein capped silver nanoparticles. Bioresour Technol.

[46] Narayanan KB, Sakthivel N (2013). Mycocrystallization of gold ions by the fungus *Cylindrocladium floridanum*. World J Microbiol Biotechnol.

[47] Narayanan KB, Sakthivel N (2011). Synthesis and characterization of nano-gold composite using *Cylindrocladium floridanum* and its heterogeneous catalysis in the degradation of 4-nitrophenol. J Hazard Mat.

[48] Sheikhloo Z, Salouti M, Katiraee F (2011). Biological synthesis of gold nanoparticles by fungus *Epicoccum nigrum*. J Clust Sci.

[49] Govender Y, Riddin T, Gericke M, Whiteley CG (2009). Bioreduction of platinum salts into nanoparticles: a mechanistic perspective. Biotechnol Lett.

[50] Rai M, Yadav A, Gade A (2009). Silver nanoparticles as a new generation of antimicrobials. Biotechnol Adv.

[51] Ahmad A, Mukherjee P, Senapat S, Mandal D, Khan MI, Kumar R, Sastry M (2003). Extracellular biosynthesis of silver nanoparticles using the fungus *Fusarium oxysporum*. Coll Surf B Biointerfaces.

[52] Ahmed A, Senapati S, Khan MI, Kumar R, Sastry M (2003). Extracellular biosynthesis of monodisperse gold nanoparticles by a novel extremophilic actinomycete, *Thermomonospora* sp. Langmuir.

[53] Mandal D, Bolander ME, Mukhopadhyay D, Sarkar G, Mukherjee P (2006). The use of microorganisms for the formation of metal nanoparticles and their application. Appl Microbiol Biotechnol.

[54] Mukherjee P, Senapati S, Mandal D, Ahmad A, Khan MI, Kumar R, Sastry M (2002). Extracellular synthesis of gold nanoparticles by the fungus *Fusarium oxysporum*. Chembiochem.

[55] Sanyal A, Rautaray D, Bansal V, Ahmad A, Sastry M (2005). Heavy-metal remediation by a fungus as a means of production of lead and cadmium carbonate crystals. Langmuir.

[56] Rautaray D, Sanyal A, Adyanthaya SD, Ahmad A, Sastry M (2004). Biological synthesis of strontium carbonate crystals using the fungus *Fusarium oxysporum*. Langmuir.

[57] Ahmad A, Mukherjee P, Senapat S, Mandal D, Khan MI, Kumar R, Sastry M (2003). Extracellular biosynthesis of silver nanoparticles using the fungus *Fusarium oxysporum*. Coll Surf B Biointerfaces.

[58] Ahmad A, Mukherjee P, Mandal D, Senapati S, Khan MI, Kumar R, Sastry M (2002). Enzymemediated extracellular synthesis of CdS nanoparticles by the fungus, *Fusarium oxysporum*. J Am Chem Soc.

[59] Bansal V, Rautaray D, Bharde A, Ahire K, Sanyal A, Ahmad A, Sastry M (2005). Fungus-mediated biosynthesis of silica and titania particles. J Mater Chem.

[60] Bansal V, Poddar P, Ahmad A, Sastry M (2006). Room-temperature biosynthesis of ferroelectric barium titanate nanoparticles. J Am Chem Soc.

[61] Bansal V, Rautaray D, Ahmad A, Sastry M (2004). Biosynthesis of zirconia nanoparticles using the fungus *Fusarium oxysporum*. J Mater Chem.

[62] Sawle BD, Salimath B, Deshpande R, Bedre MD, Prabhakar BK, Venkataraman A (2008). Biosynthesis and stabilization of Au and Au-Ag alloy nanoparticles by fungus, *Fusarium semitectum*. Sci Tech Adv Mater.

[63] SathishKumar K, Amutha R, Arumugam P, Berchmans S (2011). Synthesis of gold nanoparticles: an ecofriendly approach using *Hansenula anomala*. ACS Appl Mater Interfaces.

[64] Kumar SK, Peter YA, Nadeau JL (2008). Facile biosynthesis, separation and conjugation of gold nanoparticles to doxorubicin. Nanotechnology.

[65] Mishra AN, Bhadaurla S, Singh Gaur M, Pasricha R (2010). Extracellular microbial synthesis of gold nanoparticles using fungus *Hormoconis resinae*. JOM.

[66] Castro Longoria E, Vilchis Nestor AR, Avalos Borja M (2011). Biosynthesis of silver, gold and bimetallic nanoparticles using the filamentous fungus *Neurospora crassa*. Coll Surf B Biointerfaces.

[67] Shahverdi AR, Minaeian S, Shahverdi HR, Jamalifar H, Nohi AA (2007). Rapid synthesis of silver nanoparticles using culture supernatants of enterobacteria: a novel biological approach. Process Biochem.

[68] Ma Y, Li N, Yang C, Yang X (2005). One-step synthesis of amino-dextran protected gold and silver nanoparticles and its application in biosensors. Anal Bioanal Chem.

[69] Mishra A, Tripathy SK, Wahab R, Jeong SH, Hwang I, Yang YB, Kim YS, Shin HS, Yun SII (2011). Microbial synthesis of gold nanoparticles using the fungus *Penicillium brevicompactum* and their cytotoxic effects against mouse mayo blast cancer C_2_C_12_ cells. Appl Microbiol Biotechnol.

[70] Kathiresan K, Manivannan S, Nabeel M, Dhivya B (2009). Studies on silver nanoparticles synthesized by a marine fungus, *Penicillium fellutanum* isolated from coastal mangrove sediment. Coll Surf B Biointerfaces.

[71] Mishra A, Tripathy SK, Yuna SI (2012b) Fungus mediated synthesis of gold nanoparticles and their conjugation with genomic DNA isolated from *Escherichia coli* and *Staphylococcus aureus*. Process Biochem 47:701–711

[72] Du L, Xian L, Feng JX (2011). Rapid extra-/intracellular biosynthesis of gold nanoparticles by the fungus *Penicillium* sp. J Nanopart Res.

[73] Sanghi R, Verma P, Puri S (2011). Enzymatic formation of gold nanoparticles using *Phanerochaete chrysosporium*. Adv Chem Eng Sci.

[74] Birla SS, Tiwari VV, Gade AK, Ingle AP, Yadav AP, Rai MK (2009). Fabrication of silver nanoparticles by *Phoma glomerata* and its combined effect against *Escherichia coli*, *Pseudomonas aeruginosa* and *Staphylococcus aureus*. Lett Appl Microbiol.

[75] Vigneshwaran N, Kathe A (2007). Silver-protein (core-shell) nanoparticle production using spent mushroom substrate. Langmuir.

[76] Das SK, Dickinson C, Laffir F, Brougham DF, Marsili E (2012). Synthesis, characterization and catalytic activity of gold nanoparticles biosynthesized with *Rhizopus oryzae* protein extract. Green Chem.

[77] Sen K, Sinha P, Lahiri S (2011). Time dependent formation of gold nanoparticles in yeast cells: a comparative study. Biochem Eng J.

[78] Kowshik M, Deshmukh N, Vogel W, Urban J, Kulkarni SK, Paknikar KM (2002). Microbial synthesis of semiconductor CdS nanoparticles, their characterization, and their use in the fabrication of an ideal diode. Biotechnol Bioeng.

[79] Krumov N, Oder S, Perner Nochta I, Angelov A, Posten C (2007). Accumulation of CdS nanoparticles by yeasts in a fed-batch bioprocess. J Biotechnol.

[80] Narayanan KB, Sakthivel N (2011). Facile green synthesis of gold nanostructures by NADPH-dependent enzyme from the extract of *Sclerotium rolfsii*. Coll Surf A Physicochem Eng Asp.

[81] Mukherjee P, Roy M, Mandal BP, Dey GK, Mukherjee PK, Ghatak J, Tyagi AK, Kale SP (2008). Green synthesis of highly stabilized nanocrystalline silver particles by a non-pathogenic and agriculturally important fungus T. asperellum. Nanotechnol.

[82] Maliszewska I, Aniszkiewicz Ł, Sadowski Z (2009). Biological synthesis of gold nanostructures using the extract of *Trichoderma koningii*. Acta Phys Polon A.

[83] Maliszewska I (2013). Microbial mediated synthesis of gold nanoparticles: preparation, characterization and cytotoxicity studies. Dig J Nanomater Bios.

[84] Vahabi K, Mansoori GA, Karimi S (2011). Biosynthesis of silver nanoparticles by fungus *Trichoderma reesei* (a route for large-scale production of AgNPs). Insci J.

[85] Fayaz M, Tiwary CS, Kalaichelvan PT, Venkatesan R (2010). Blue orange light emission from biogenic synthesized silver nanoparticles using *Trichoderma viride*. Coll Surf B Biointerfaces.

[86] Ramanathan R, Field MR, O’Mullane AP, Smooker PM, Bhargava SK, Bansal V (2013). Aqueous phase synthesis of copper nanoparticles: a link between heavy metal resistance and nanoparticle synthesis ability in bacterial systems. Nanoscale.

[87] Mukherjee P, Ahmad A, Mandal D, Senapati S, Sainkar SR, Khan MI, Ramani R, Parischa R, Ajaykumar PV, Alam M, Sastry M, Kumar R (2001). Bioreduction of AuCl_4_-ions by the fungus, *Verticillium* sp. and surface trapping of the gold nanoparticles formed. Angew Chem Int Edu.

[88] Philip D (2009). Biosynthesis of Au, Ag and Au-Ag nanoparticles using edible mushroom extract. Spectrochim Acta Mol Biomol Spectrosc.

[89] Agnihotri M, Joshi S, Ravikumar A, Zinjarde S, Kulkarni S (2009). Biosynthesis of gold nanoparticles by the tropical marine yeast *Yarrowia lipolytica* NCIM 3589. Mater Lett.

[90] Pimprikar PS, Joshi SS, Kumar AR, Zinjarde SS, Kulkarni SK (2009). Influence of biomass and gold salt concentration on nanoparticle synthesis by the tropical marine yeast *Yarrowia lipolytica* NCIM 3589. Coll Surf B Biointerfaces.

[91] Lloyd JR (2003). Microbial reduction of metals and radionuclides. FEMS Microbial Rev.

[92] Medentsev AG, Alimenko VK (1998). Naphthoquinone metabolites of the fungi. Photochemistry.

[93] Baker RA, Tatum JH (1998). Novel anthraquinones from stationary cultures of *Fusarium oxysporum*. J Ferment Bioeng.

[94] Durán N, Teixeira MFS, De Conti R, Esposito E (2002). Ecological-friendly pigments from fungi. Crit Rev Food Sci Nutr.

[95] Bell AA, Wheeler MH, Liu J, Stipanovic RD, Puckhaber LS, Orta H (2003). United States Department of Agriculture—Agricultural Research Service studies on polyketide toxins of *Fusarium oxysporum f* sp *Vasinfectum*: potential targets for disease control. Pest Manag Sci.

[96] Chen JC, Lin ZH, Ma XX (2003). Evidence of the production of silver nanoparticles via pretreatment of *Phoma* sp.3.2883 with silver nitrate. Lett Appl Microbiol.

[97] Jebali A, Ramezani F, Kazemi B (2011). Biosynthesis of silver nanoparticles by *Geotricum* sp. J Clus Sci.

[98] Yong P, Rowson AN, Farr JPG, Harris IR, Mcaskie LE (2002). Bioaccumulation of palladium by *Desulfovibrio desulfuricans*. J Chem Technol Biotechnol.

[99] Kowshik M, Ashtaputre S, Kulkani SK, Parknikar KMM (2003). Extracellular synthesis of silver nanoparticles by a silver-tolerant yeast strain MKY3. Nanotechnology.

[100] Subramanian M, Alikunhi MN, Kathiresan K (2010). *In vitro* synthesis of silver nanoparticles by marine yeasts from coastal mangrove sediment. Adv Sci Lett.

[101] Jain N, Bhargava A, Majumdar S, Tarafdar JC, Panwar J (2011). Extracellular biosynthesis and characterization of silver nanoparticles using *Aspergillus flavus* NJP08: a mechanism perspective. Nanoscale.

[102] Vigneshwaran N, Ashtaputre NM, Varadarajan PV, Nachane RP, Paralikar KM, Balasubramanya RH (2007). Biological synthesis of silver nanoparticles using the fungus *Aspergillus flavus*. Mat Lett.

[103] Senapati S, Mandal D, Ahmad A (2004). Fungus mediated synthesis of silver nanoparticles: a novel biological approach. Ind J Phys A.

[104] Gericke M, Pinches A (2006). Biological synthesis of metal nanoparticles. Hydrometallurgy.

[105] Sneha K, Sathishkumar M, Mao J, Kwak IS, Yun YS (2010). Corynebacterium glutamicum-mediated crystallization of silver ions through sorption and reduction processes. Chem Eng J.

[106] Mukherjee P, Ahmad A, Mandal D, Senapati S, Sainkar SR, Khan MI, Parischa R, Ajaykumar PV, Alam M, Kumar R, Sastry M (2001). Fungus mediated synthesis of silver nanoparticles and their immobilization in the mycelial matrix: a novel biological approach to nanoparticle synthesis. Nano Lett.

[107] Daniel MC, Astruc D (2004). Gold nanoparticles: assembly, supramolecular chemistry, quantum-size-related properties, and applications toward biology, catalysis, and nanotechnology. Chem Rev.

[108] Murphy CJ, Gole AM, Stone JW, Sisco PN, Alkilany AM, Goldsmith EC, Baxter SC (2008). Gold nanoparticles in biology: beyond toxicity to cellular imaging. Acc Chem Res.

[109] Andreescu D, Kumar Sau K, Goia DV (2006). Stabilizer-free nanosized gold sols. J Coll Interface Sci.

[110] Pimpang P, Choopun S (2011). Monodispersity and stability of gold nanoparticles stabilized by using polyvinyl alcohol. Chiang Mai J Sci.

[111] Sanghi R, Verma P, Puri S (2011). Enzymatic formation of gold nanoparticles using *Phanerochaete chrysosporium*. Adv Chem Eng Sci.

[112] Gupta S, Bector S (2013). Biosynthesis of extracellular and intracellular gold nanoparticles by *Aspergillus fumigatus* and *A. flavus*. Anton Leeuw.

[113] Das SK, Liang J, Schmidt M, Laffir F, Marsili E (2012). Biomineralization mechanism of gold by zygomycete fungi *Rhizopous oryzae*. ACS Nano.

[114] Lim HA, Mishra A, Yun SI (2011). Effect of pH on the extra cellular synthesis of gold and silver nanoparticles by *Saccharomyces cerevisae*. J Nanosci Nanotechnol.

[115] Mishra A, Tripathy S, Yun SI (2011). Bio-synthesis of gold and silver nanoparticles from Candida guilliermondii and their antimicrobial effect against pathogenic bacteria. J Nanosci Nanotechnol.

[116] Soni N, Prakash S (2012). Synthesis of gold nanoparticles by the fungus *Aspergillus niger* and its efficacy against mosquito larvae. Rep Parasitolo.

[117] Castro Longoria E, Moreno Velázquez SD, Vilchis Nestor AR, Arenas Berumen E, Avalos Borja M (2012). Production of platinum nanoparticles and nanoaggregates using *Neurospora crassa*. J Microbiol Biotechnol.

[118] Bharde A, Rautaray D, Bansal V, Ahmad A, Sarkar I, Yusuf SM, Sanyal M, Sastry M (2006). Extracellular biosynthesis of magnetite using fungi. Small.

[119] Zare B, Babaie S, Setayesh N, Shahverdi AR (2013). Isolation and characterization of a fungus for extracellular synthesis of small selenium nanoparticles. Nanomed J.

[120] Dameron CT, Reese RN, Mehra RK, Kortan AR, Carroll PJ, Steigerwald ML, Winge BLE, Winge DR (1989). Biosynthesis of cadmium sulphide quantum semiconductor crystallites. Nature.

[121] Agrios GN (2005). Plant pathology.

[122] Fernandez RG, Prats E, Jorrin Novo JV (2010). Proteomics of plant pathogenic fungi. J Biomed Biotechnol.

[123] Ocsoy I, Paret ML, Ocsoy MA, Kunwar S, Chen T, You M, Tan W (2013). Nanotechnology in plant disease management: DNA-directed silver nanoparticles on graphene oxide as an antibacterial against *Xanthomonas perforans*. ACS Nano.

[124] Chambers VW, Proctor CM, Kabler PW (1962). Bactericidal effects of low concentrations of silver. J Am Water Works Assoc.

[125] Kim JH (2004). Nano silver chemotherapeutic agents and its applications. News Inf Chem Eng.

[126] Jo YK, Kim BH, Jung G (2009). Antifungal activity of silver ions and nanoparticles on phytopathogenic fungi. Plant Dis.

[127] Davies RL, Etris SF (1997). The development and functions of silver in water purification and disease control. Catal Today.

[128] Kim SW, Jung JH, Lamsal K, Kim YS, Min JS, Lee YS (2012). Antifungal effects of silver nanoparticles (AgNPs) against various plant pathogenic fungi. Mycobiology.

[129] Min JS, Kim KS, Kim SW, Jung JH, Lamsal K, Kim SB, Jung M, Lee YS (2009). Effects of colloidal silver nanoparticles on sclerotium-forming phytopathogenic fungi. Plant Pathol J.

[130] Kim H, Kang H, Chu G, Byun G (2008). Antifungal effectiveness of nanosilver colloid against rose powdery mildew in greenhouses. Solid State Phenom.

[131] Aguilar-Méndez AM, Martín-martínez ES, Ortega-Arroyo L, Cobián-Portillo G, Sánchez-Espíndola E (2010). Synthesis and characterization of silver nanoparticles: effect on phytopathogen *Colletotrichum gloesporioides*. J Nanopart Res.

[132] Das SK, Das AR, Guha AK (2009). Gold nanoparticles: microbial synthesis and application in water hygiene management. Langmuir.

[133] Salata OV (2004). Applications of nanoparticles in biology and medicine. J Nanobiotechnol.

[134] Yadav A, Kon K, Kratosova G, Duran N, Ingle AP, Rai M (2015). Fungi as an efficient mycosystem for the synthesis of metal nanoparticles: progress and key aspects of research. Biotechnol Lett.

[135] Khan MM, Kalathil S, Han TH, Lee J, Cho MH (2013). Positively charged gold nanoparticles synthesized by electrochemically active biofilm—a biogenic approach. J Nanosci Nanotechnol.

[136] Gajbhiye MB, Kesharwani JG, Ingle AP, Gade AK, Rai MK (2009). Fungus mediated synthesis of silver nanoparticles and its activity against pathogenic fungi in combination of fluconazole. Nanomedicine.

[137] Saravanan M, Nanda A (2010). Extracellular synthesis of silver bionanoparticles from *Aspergillus clavatus* and its antimicrobial activity against MRSA and MRSE. Colloids Surf B: Biointerfaces.

[138] Gade AK, Bonde P, Ingle AP, Marcato PD, Duran N, Rai MK (2008). Exploitation of *Aspergillus niger* for synthesis of silver nanoparticles. J Biobased Mater Bioener.

[139] Sundaramoorthi C, Kalaivani M, Mathews DM, Palanisamy S, Kalaiselvan V, Rajasekaran A (2009). Biosynthesis of silver nanoparticles from *Aspergillus niger *and evaluation of its wound healing activity in experimental rat model. Int J Pharm Tech Res.

[140] Ingle A, Gade A, Pierrat S, Sönnichsen C, Rai M (2008). Mycosynthesis of silver nanoparticles using the fungus Fusarium acuminatum and its activity against some human pathogenic bacteria. Curr Nanosci.

[141] Durán N, Marcato PD, De S, Gabriel IH, Alves OL, Esposito E (2007). Antibacterial effect of silver nanoparticles produced by fungal process on textile fabrics and their effluent treatment. J Biomed Nanotechnol.

[142] El-Rafie MH, Mohamed AA, Shaheen THI, Hebeish A (2010). Antimicrobial effect of silver nanoparticles produced by fungal process on cotton fabrics. Carbo Polym.

[143] Namasivayam SKR, Avimanyu (2011). Silver nanoparticle synthesis from *Lecanicillium lecanii* and evalutionary treatment on cotton fabrics by measuring their improved antibacterial activity with antibiotics against *Staphylococcus aureus* (ATCC 29213) and *E. coli* (ATCC 25922) strains. Int J Pharm Pharm Sci.

[144] Chowdhury S, Basu A, Kundu S (2014). Green synthesis of protein capped silver nanoparticles from phytopathogenic fungus *Macrophomina phaseolina* (Tassi) Goid with antimicrobial properties against multidrug-resistant bacteria. Nano Res Lett.

[145] Du L, Xu Q, Huang M, Xian L, Feng JX (2015). Synthesis of small silver nanoparticles under light radiation by fungus *Penicillium oxalicum* and its application for the catalytic reduction of methylene blue. Mat Chem Phy.

[146] Singh D, Rathod V, Ninganagouda S, Hiremath J, Singh AK, Mathew J (2014). Optimization and characterization of silver nanoparticle by endophytic fungi *Penicillium* sp. isolated from *Curcuma longa* (Turmeric) and application studies against MDR *E. coli* and *S. aureus*. Bioinorg Chem App.

[147] Thirumurugan G, Shaheedha SM, Dhanaraju MD (2009). In vitro evaluation of anti-bacterial activity of silver nanoparticles synthesised by using *Phytophthora infestans*. Int J Chem Tech R.

[148] Devika R, Elumalai S, Manikandan E, Eswaramoorthy D (2012). Biosynthesis of silver nanoparticles using the fungus *Pleurotus ostreatus* and their antibacterial activity. Open Access Sci Rep.

[149] Kim SW, Kim KS, Lamsal K, Kim YJ, Kim SB, Jung M, Sim SJ, Kim HS, Chang SJ, Kim JK, Lee YS (2009). An in vitro study of the antifungal effect of silver nanoparticles on oak wilt pathogen *Raffaelea* sp. J Microbiol Biotechnol.

[150] Ray S, Sarkar S, Kundu S (2011). Extracellular biosynthesis of silver nanoparticles using the mycorrhizal mushroom *Tricholoma crassum* (BERK.) Sacc.: its antimicrobial activity against pathogenic bacteria and fungus, including multidrug resistant plant and human bacteria. Dig J Nanomater Bios.

[151] Fayaz AM, Balaji K, Girilal M, Kalaichelvan PT, Venkatesan R (2009). Mycobased synthesis of silver nanoparticles and their incorporation into sodium alginate films for vegetable and fruit preservation. J Agric Food Chem.

[152] Bhargava A, Jain N, Gangopadhyay S, Panwar J (2015). Development of gold nanoparticle-fungal hybrid based heterogeneous interface for catalytic applications. Pro Biochem.

[153] Kumar SA, Abyaneh MK, Gosavi SW, Kulkarni SK, Pasricha R, Ahmad A, Khan MI (2007). Nitrate reductase-mediated synthesis of silver nanoparticles from AgNO_3_. Biotechnol Lett.

[154] Morones JR, Elechiguerra JL, Camacho A, Holt K, Kouri JB, Ramfrez JT, Yacaman MJ (2005). The bactericidal effect of silver nanoparticles. Nanotechnology.

[155] Raheman F, Deshmukh S, Ingle A, Gade A, Rai M (2011). Silver nanoparticles: novel antimicrobial agent synthesized from an endophytic fungus *Pestalotia* sp. isolated from leaves of *Syzygium cumini* (L). Nano Biomed Eng.

[156] Spasova M, Manolova N, Naydenov M, Kuzmanova J (2011). Electrospun biohybrid materials for plant biocontrol containing chitosan and *Trichoderma viride* spores. J Bioact Compat Polym.

[157] Shao L, Gao Y, Feng Y (2011). Semiconductor quantum dots for biomedical applications. Sensors.

[158] Kumar AS, Ansary AA, Ahmad A, Khan MI (2007). Extracellular biosynthesis of CdSe quantum dots by the fungus *Fusarium oxysporum*. J Biomed Nanotechnol.

[159] Bakar NA, Salleh MM, Umar AA, Yahaya M (2011). The detection of pesticides in water using ZnCdSe quantum dot films. Adv Nat Sci Nanosci Nanotechnol.

